# The Tarantula Toxin ω-Avsp1a Specifically Inhibits Human Ca_V_3.1 and Ca_V_3.3 via the Extracellular S3-S4 Loop of the Domain 1 Voltage-Sensor

**DOI:** 10.3390/biomedicines10051066

**Published:** 2022-05-04

**Authors:** Volker Herzig, Yong-Cyuan Chen, Yanni K.-Y. Chin, Zoltan Dekan, Yu-Wang Chang, Hui-Ming Yu, Paul F. Alewood, Chien-Chang Chen, Glenn F. King

**Affiliations:** 1 Institute for Molecular Bioscience, The University of Queensland, Brisbane, QLD 4072, Australia; yanni.chin@imb.uq.edu.au (Y.K.-Y.C.); z.dekan@imb.uq.edu.au (Z.D.); p.alewood@imb.uq.edu.au (P.F.A.); 2 Centre for Bioinnovation, University of the Sunshine Coast, Sippy Downs, QLD 4556, Australia; 3 School of Science, Technology and Engineering, University of the Sunshine Coast, Sippy Downs, QLD 4556, Australia; 4 Institute of Biomedical Sciences, Academia Sinica, Taipei 11529, Taiwan; joyoyo@ibms.sinica.edu.tw (Y.-C.C.); awanya@ibms.sinica.edu.tw (Y.-W.C.); 5 Centre for Advanced Imaging, The University of Queensland, Brisbane, QLD 4072, Australia; 6 Genomics Research Center, Academia Sinica, Taipei 11529, Taiwan; hmyu@gate.sinica.edu.tw; 7 Australian Research Council Centre of Excellence for Innovations in Peptide and Protein Science, The University of Queensland, St. Lucia, QLD 4072, Australia

**Keywords:** voltage-gated calcium channel, Ca_V_3 subtype, T-type, venom peptide synthesis, theraphosid spider, tarantula, peptide structure

## Abstract

Inhibition of T-type calcium channels (Ca_V_3) prevents development of diseases related to cardiovascular and nerve systems. Further, knockout animal studies have revealed that some diseases are mediated by specific subtypes of Ca_V_3. However, subtype-specific Ca_V_3 inhibitors for therapeutic purposes or for studying the physiological roles of Ca_V_3 subtypes are missing. To bridge this gap, we employed our spider venom library and uncovered that *Avicularia spec.* (“Amazonas Purple”, Peru) tarantula venom inhibited specific T-type Ca_V_ channel subtypes. By using chromatographic and mass-spectrometric techniques, we isolated and sequenced the active toxin ω-Avsp1a, a C-terminally amidated 36 residue peptide with a molecular weight of 4224.91 Da, which comprised the major peak in the venom. Both native (4.1 μM) and synthetic ω-Avsp1a (10 μM) inhibited 90% of Ca_V_3.1 and Ca_V_3.3, but only 25% of Ca_V_3.2 currents. In order to investigate the toxin binding site, we generated a range of chimeric channels from the less sensitive Ca_V_3.2 and more sensitive Ca_V_3.3. Our results suggest that domain-1 of Ca_V_3.3 is important for the inhibitory effect of ω-Avsp1a on T-type calcium channels. Further studies revealed that a leucine of T-type calcium channels is crucial for the inhibitory effect of ω-Avsp1a.

## 1. Introduction

Dynamic changes in the intracellular calcium concentration ([Ca]_i_) are used to regulate various biological processes [1], with abnormal homeostasis of [Ca]_i_ leading to a diverse spectrum of diseases [2]. Both high-voltage-activated calcium channels (Ca_V_1 and Ca_V_2 subtypes) and low-voltage-activated calcium channels (Ca_V_3 subtypes) are involved in regulation of [Ca]_i_ in both excitable and non-excitable cells. Ca_V_3 channels (also known as T-type channels) are expressed in a variety of tissues, including the heart, nervous system, smooth muscle, kidney, sperm, and endocrine organs. The differences in biophysical properties and pharmacological responses recorded for T-type currents from various cell types are partially due to differences in tissue-specific expression of the three Ca_V_3 subtypes —Ca_V_3.1, Ca_V_3.2 and Ca_V_3.3 [3]. For example, Ca_V_3.1 contributes to low-threshold calcium spikes in the thalamus [4], whereas Ca_V_3.2 mediates coronary vessel relaxation in mice [5].

Increased T-type calcium currents are found in various pathological conditions due to upregulation of specific Ca_V_3 subtypes. For example, Ca_V_3.1 mRNA and protein are upregulated in vascular smooth muscle cells during injury-induced neointimal formation. Knockout of Ca_V_3.1, but not Ca_V_3.2, prevents neointimal formation in injured carotid arteries [6]. Mice lacking Ca_V_3.1 are also resistant to high-fat diet-induced weight gain [7] but have fragmented sleep and reduced slow-wave sleep [8,9]. In contrast, Ca_V_3.2 mRNA and currents are upregulated in cardiomyocytes in a mouse model of pressure-overload induced cardiac hypertrophy. Knockout of Ca_V_3.2, but not Ca_V_3.1, inhibits pathological cardiac hypertrophy [10]. In addition, Ca_V_3.2 but not Ca_V_3.1 are highly expressed in dorsal root ganglion (DRG) sensory neurons [11]. Enhanced Ca_V_3.2 activity is seen in DRG neurons during neuropathic and inflammatory pain [12]. Silencing or knockout of Ca_V_3.2, but not Ca_V_3.1, inhibits the development of neuropathic, inflammatory or muscle pain [11,12,13]. These knockout animal studies indicate the potential therapeutic benefit of Ca_V_3 channel blockers. However, Ca_V_3.1 and Ca_V_3.2 play different, even opposite, roles in the cardiovascular and nervous systems [14]. For example, Ca_V_3.1 may have a protective role in pathological cardiac hypertrophy [15], whereas Ca_V_3.2 is involved in the development of pathological cardiac hypertrophy [10]. Thus, for treating some of these Ca_V_3-related diseases, it will be essential to develop subtype-specific inhibitors to avoid undesired side-effects.

Given the important role of Ca_V_ channels in many physiological processes, venomous animals have evolved Ca_V_ channel inhibitors to interfere with normal physiological processes for assisting with prey capture or defence against predators [16,17]. Spider venoms are well known for containing inhibitors of both insect and vertebrate Ca_V_ channels [18,19,20]. Therefore, the aim of this study was to identify potential Ca_V_3-subtype specific modulators from our in-house collection of arachnid venoms. In preliminary experiments, we screened a panel of 1 scorpion and 26 spider venoms (Supplementary Table S1) for their activity against human Ca_V_3.1, Ca_V_3.2, and Ca_V_3.3 using patch-clamp electrophysiology The venom of an uncharacterized Peruvian tarantula (*Avicularia spec.*) was found to specifically inhibit Ca_V_3.1 and Ca_V_3.3 subtypes. This venom was therefore chosen for further characterisation with the aim of isolating and characterising the active venom component (Supplementary Figure S1).

## 2. Materials and Methods

### 2.1. Venoms

All arachnid venoms used for this study (for details see Supplementary Table S1) were isolated by light electrical stimulation as previously described [21], then lyophilised and stored at −20 °C prior to use.

### 2.2. Reversed-Phase High Pressure Liquid Chromatography

*Avicularia spec.* (“Amazonas Purple”, Peru) venom was fractionated via reversed-phase high-performance liquid chromatography (RP-HPLC) on a Prominence HPLC system (Shimadzu Scientific Instruments, Rydalmere, NSW, Australia). Venom was loaded onto a C_18_ RP-HPLC column (Phenomenex Jupiter; 250 Å, 250 × 4.6 mm, 5 μm), and fractionated using the following gradient: 5% solvent B (0.043% trifluoroacetic acid (TFA) in 90% acetonitrile) in solvent A (0.05% TFA in H_2_O) for 5 min, 5–20% solvent B over 5 min, 20–40% solvent B over 40 min, then 40–80% solvent B for 5 min (flow rate 1 mL/min). The peak containing the active peptide (termed ω-Avsp1a, see Section 3.1 for details) was further purified by Hydrophilic Interaction Liquid Chromatography (HILIC) using a Grace VisionHT HILIC column (150 × 4.6 mm, 5 μm) on a Shimadzu Prominence HPLC system with a flow rate of 1 mL/min and using the same solvents as for the initial RP-HPLC run. After 3 min at 95% solvent B, a linear gradient of 95–75% B was run over 20 min, followed by a decrease from 75 to 5% B within 2 min.

### 2.3. Mass Spectrometry

All molecular masses were determined by using matrix-assisted laser desorption/ionization time-of-flight mass spectrometry (MALDI-TOF MS) using a Model 4700 Triple TOF mass spectrometer (Applied Biosystems, Foster City, CA, USA). Toxin samples were mixed 1:1 (*v*:*v*) with α-cyano-4-hydroxy-cinnamic acid matrix (7 mg/mL in 50/50 acetonitrile/H_2_O with 5% formic acid) and MALDI-TOF spectra were acquired in positive reflector mode. All reported molecular masses unless otherwise stated refer to the monoisotopic uncharged peptide.

### 2.4. N-Terminal Edman Sequencing

ω-Avsp1a was solubilised in 25 mM ammonium bicarbonate, reduced using dithiothreitol (25 mM) at 56 °C for 30 min, then alkylated using iodoacetamide (55 mM) at room temperature for 30 min. Fully reduced and alkylated Avsp1a was then purified via RP-HPLC using a Zorbax 300SB-C18 column (3 Å, 150 × 3 mm). The purified reduced/alkylated Avsp1a was then loaded onto a precycled Biobrene disc and N-terminal sequencing via Edman degradation was performed by the Australian Proteome Analysis Facility (Sydney, NSW, Australia) using an Applied Biosystems 494 Procise Protein Sequencer.

### 2.5. C-Terminal Sequencing Using Carboxypeptidase Y

The C-terminal sequence of native Avsp1a was determined by incubation with carboxypeptidase Y (CPY) according to the method previously described [22]. In short, 4.4 μg of native Avsp1a was digested with CPY (2 ng) for various time points (1, 2, 5, 15, 30, 60, 120, and 240 min), before the reaction was quenched by adding 1 μL of 1% formic acid. The resulting fragments were then analysed via MALDI–TOF MS as described in Section 2.3.

### 2.6. Chemical Synthesis of ω-Avsp1a

ω-Avsp1a was assembled manually using standard Fmoc solid-phase peptide synthesis protocols. The peptide was assembled on 0.1 mmol scale and Fmoc-Rink-amide polystyrene resin (loading 0.67 mmol/g). Side chains of Fmoc-amino acids were protected as follows: Arg(Pbf), Asp(tBu), Cys(Trt), Glu(tBu), His(Trt), Lys(Boc), Ser(tBu), Thr(tBu) and Trp(Boc). Fmoc deprotections were accomplished by treatment with 30% piperidine/dimethlylformamide (DMF) (1 × 1.5 min then 1 × 3 min). Fmoc-amino acids (5 equiv.) were coupled in DMF using O-(1H-6-chlorobenzotriazole-1-yl)-1,1,3,3-tetramethyluronium hexafluorophosphate (HCTU) (5 equiv) and *N*,*N*-diisopropylethylamine (DIEA) (5 equiv) for 1 × 1 min then 1 × 5 min. The peptide resin was cleaved with TFA/triisopropylsilane (TIPS)/H_2_O (38:1:1) for 2 h with stirring at room temperature. Following evaporation of most of the cleavage solution, the product was precipitated and washed with cold diethylether then lyophilised from 0.05% TFA in ACN/H_2_O (1:1). The crude linear peptide with correct mass for reduced ω-Avsp1a was purified via preparative RP-HPLC.

The purified reduced peptide was oxidatively folded in 0.33 M ammonium acetate, 2 M guanidine hydrochloride (pH 8.0), in the presence of 10- or 100-fold molar equivalents of oxidised and reduced glutathione respectively, stirring at 4 °C for 3 days. The single major product was isolated by preparative RP-HPLC, then co-eluted with native ω-Avsp1a on analytical RP-HPLC to confirm that the correct fold had been achieved.

### 2.7. Cell Culture and Transient Expression

Human embryonic kidney (HEK) 293 cells were grown in Dulbecco’s modified Eagle’s medium (DMEM) containing 5% fetal bovine serum and penicillin/streptomycin at 37 °C in a 5% CO_2_ incubator. Cells were transiently transfected with 3 μg plasmid DNA using the calcium-phosphate method. Plasmids of Cav3.1, Cav3.2 or Cav3.3 were separately transfected into three individual plates of HEK-293 cells. After transfection, cells were incubated at 37 °C for 48 h prior to whole-cell voltage-clamp recordings.

### 2.8. Electrophysiology

Patch pipettes with a tip resistance of 2.8~3.5 MΩ were pulled from borosilicate glass capillary tubes (Warner Instruments, Holliston, MA, USA) using a P-97 Flaming-Brown type micropipette puller (Sutter Instrument, Novato, CA, USA). Ionic currents were recorded using an Axon Multiclamp 700B microelectrode amplifier (Molecular Devices, San Jose, CA, USA). The sampling frequency for acquisition was 50 kHz and data were low-pass filtered at 2 kHz. Voltage and current commands and digitisation of membrane voltages and currents were controlled using a Digidata 1440A interfaced with Clampex 10.4 (Molecular Devices, San Jose, CA, USA). Data were analysed using pCLAMP10.4 software (Molecular Devices, San Jose, CA, USA).

To measure Ca_V_3 currents, cells were incubated with the following bath solution (in mM): 145 TEA-Cl, 5 CaCl_2_, 3 CsCl, 1 MgCl_2_, 5 glucose, and 10 HEPES, adjusted to pH 7.4 with TEA-OH, osmolarity ~300 mOsm. The pipette solution was as follows (in mM): 130 CsCl, 20 HEPES, 10 EGTA, 5 MgCl_2_, 3 Mg-ATP, and 0.3 Tris-GTP, adjusted to pH 7.3 with CsOH, osmolarity ~310 mOsm. All recordings were performed at room temperature (21–24 °C). For Cav3.1- or Cav3.2-transfected cells, their membrane potentials were initially held at −90 mV for 20 ms and then depolarized to −30 mV for 150 ms. For Cav3.3-transfected cells, the membrane potentials were initially held at −90 mV for 20 ms and then depolarized to −20 mV for 150 ms. Application of venom fractions or synthetic peptide was via a Picospritzer III dispenser (Parker Hannifin, Hollis, NH, USA). Puffed pressure and duration were 8–10 psi and 60 s, respectively. The peak values of Cav3 currents were recorded before and after addition of venom fractions or synthetic peptide. The toxin-reduced peak amplitudes represent the changes of peak values before or after addition of the toxin. The inhibition was calculated by dividing the toxin-reduced peak amplitudes by the peak amplitudes before additions of the toxin.

### 2.9. Construction and Mutagenesis of Plasmid cDNA

All plasmid constructs were made using a QuikChange site-directed mutagenesis kit (Agilent Technologies, Santa Clara, CA, USA). After mutagenesis, constructs were verified by sequencing. Chimeric constructs were combined by using the GeneArt Seamless Cloning and Assembly Enzyme Mix (Thermo Fisher Scientific, Waltham, MA, USA).

### 2.10. Determination of Three-Dimensional (3D) Structure of ω-Avsp1a by NMR

The 3D structure of ω-Avsp1a was determined using 2D NMR spectroscopy. Lyophilised synthetic peptide was reconstituted to a final concentration of 1 mM in buffer containing 20 mM sodium phosphate at pH 6 and 5% D_2_O. All NMR spectra were recorded at 25 °C on a 600 MHz Bruker Advance II spectrometer (Billerica, MA, USA) equipped with a cryogenic probe. A combination of 2D spectra including natural abundance ^1^H-^15^N HSQC, ^1^H-^13^C HSQC, ^1^H-^1^H TOCSY and ^1^H-^1^H NOESY were acquired for resonance assignments. The spectra were processed using Topspin 3.2 (Bruker) and analysed using CcpNmr Analysis 2.4.1 [23]. Chemical shift assignments were deposited in BioMagResBank (BMRB) under the accession number 30872. The 2D ^1^H-^1^H NOESY acquired with a mixing time of 250 ms provided interproton distance restraints for the structure calculations. The spectrum was manually peak-picked and CYANA (v.3.97) was employed to automatically assign the peak list and extract distance restraints [24]. Dihedral-angle restraints were derived from chemical shifts using TALOS-N [25], with the restraint ranges for structure calculations set to twice the standard deviation. CYANA was used to calculate 200 structures based on 565 interproton distance restraints, 52 dihedral-angle restraints and 9 disulfide-bond restraints. The 30 structures with the lowest final target-function values were selected and were subjected to MolProbity analysis [26]. The 20 structures with the best stereochemical properties as judged by MolProbity were chosen to represent the structure of ω-Avsp1a. The atomic coordinates of the final structure ensemble have been deposited in the Protein Data Bank (PDB) under accession code 7LVN. A summary of the restraints used in the structure calculation and the final structure statistics are presented in Table 1.

## 3. Results

### 3.1. Toxin Isolation and Purification

**Figure 1:**
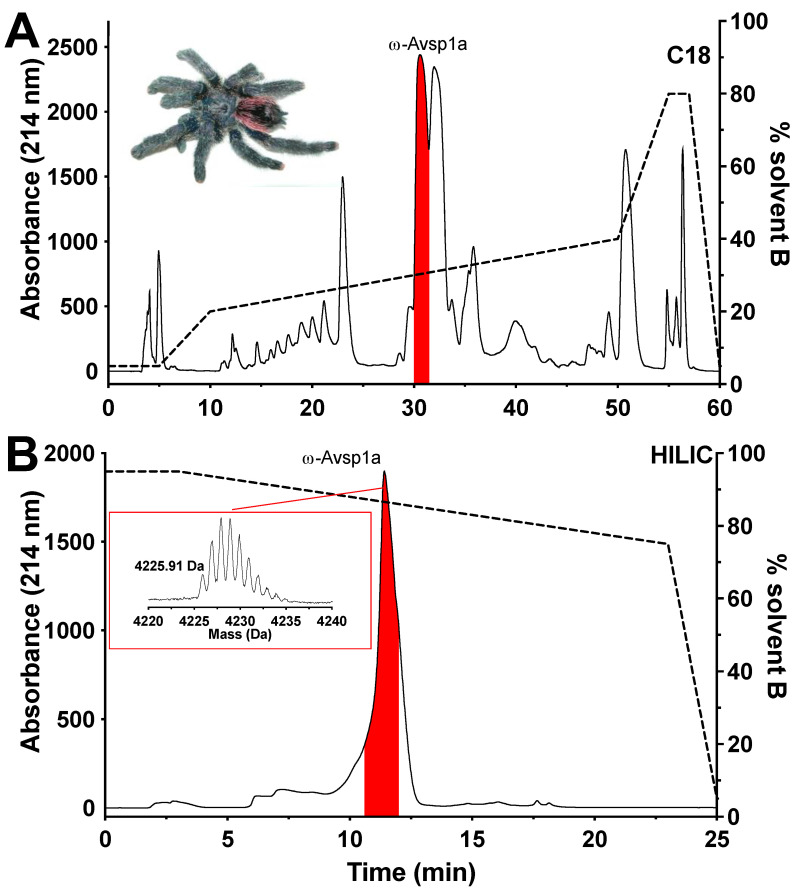
Isolation and purification of ω-Avsp1a. (**A**) Chromatogram resulting from fractionation of crude venom from *Avicularia spec.* (“Amazonas Purple”, Peru; inset shows photo of representative specimen by V.H.) using C_18_ RP-HPLC. (**B**) Chromatogram resulting from further purification of the red fraction shown in panel A using HILIC-HPLC. Inset shows a MALDI-MS spectrum of purified ω-Avsp1a with the molecular mass indicated as [M + H]^+^. The dashed line indicates the gradient of solvent B (90% ACN/0.05% TFA). The red peak contains the peptide ω-Avsp1a that is active on Ca_V_3.

An initial screen of a panel of arachnid venoms (Supplementary Table S1) revealed that crude venom (200 ng/μL) from the tarantula *Avicularia spec.* (“Amazonas Purple”, Peru) specifically inhibited Ca_V_3.1 and Ca_V_3.3 subtypes. *Avicularia spec.* venom was then fractionated via RP-HPLC, with the largest peak (highlighted in red Figure 1A) reproducing the Ca_V_3 inhibition seen with crude venom. Further purification of the active fraction by HILIC-HPLC (Figure 1B) yielded a pure peptide with a molecular mass of 4224.91 Da.

**Figure 2:**
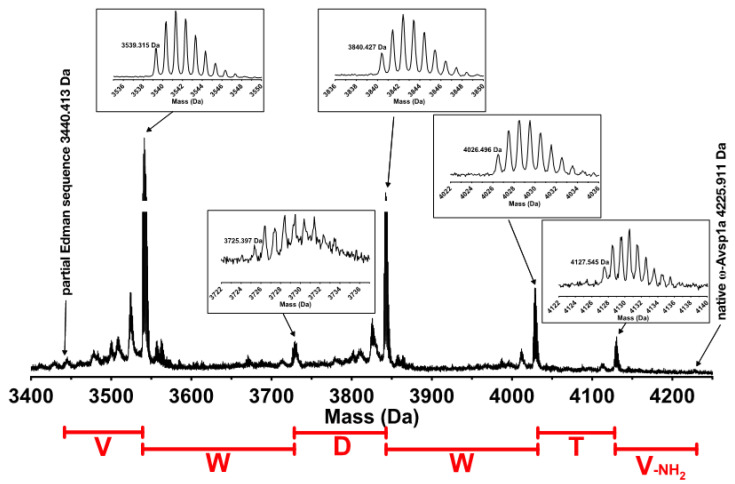
C-terminal sequencing of ω-Avsp1a after incubation with CBY for 60 min. Insets show zoomed spectra for the relevant fragments. The C-terminal sequence obtained from this data is indicated in red. All molecular masses are presented as [M + H]^+^.

N-terminal Edman sequencing of the purified active peptide yielded the sequence GD**C**HKFLGW**C**RGEPDP**CC**EHLS**C**SRKHGW**C** with the predicted molecular mass being 785.50 Da less than that observed for the native toxin. We therefore performed further C-terminal sequencing using digestion with CPY. These experiments revealed a C-terminal sequence of VWDWTV-NH_2_ (Figure 2). Together with the N-terminal sequence obtained by Edman (GDCHKFLGWCRGEPDPCCEHLSCSRKHGWC), the resulting complete sequence can be deduced as GD**C**HKFLGW**C**RGEPDP**CC**EHLS**C**SRKHGW**C**VWDWTV-NH_2_. This sequence has a calculated monoisotopic molecular mass of 4224.80 Da, only 0.11 Da less than the observed mass of the native peptide. We therefore conclude that this is the complete sequence of the peptide. Based on the rules for the rational nomenclature of venom peptides [27] and its activity on Ca_V_ channels, we propose the following toxin name: ω-theraphotoxin-Avsp1a (or ω-TRTX-Avsp1a as the official abbreviation). Henceforth, for simplification, we refer to this toxin as ω-Avsp1a.

### 3.2. Chemical Synthesis of ω-Avsp1a

**Figure 3:**
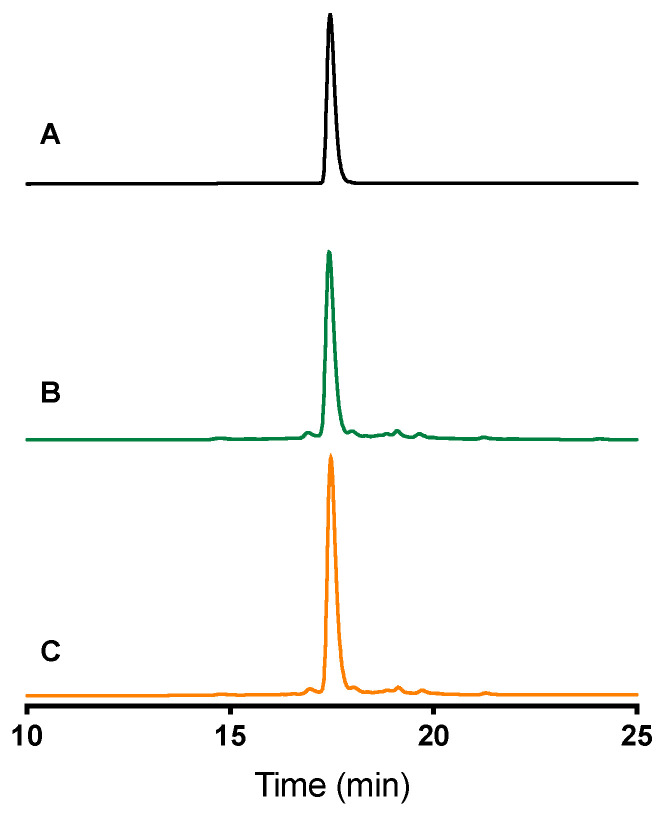
Analytical RP-HPLC chromatograms resulting from injection of (**A**) synthetic ω-Avsp1a; (**B**) native ω-Avsp1a, and (**C**) both the native and synthetic forms. The two forms co-elute, indicating that the synthetic peptide is correctly folded. Column: Thermo Hypersil C18 GOLD 100 × 2.1 mm, 3 μm, 175 Å. Conditions: 10 to 55% solvent B over 30 min, column heated at 40 °C, UV detection at 214 nm.

ω-Avsp1a was synthesised using solid-phase peptide synthesis, and oxidatively folded to yield the correct disulfide-bond isomer as confirmed by co-elution with native ω-Avsp1a (Figure 3).

### 3.3. Sub-Type Specific Inhibition of T-Type Calcium Channels by ω-Avsp1a

**Figure 4:**
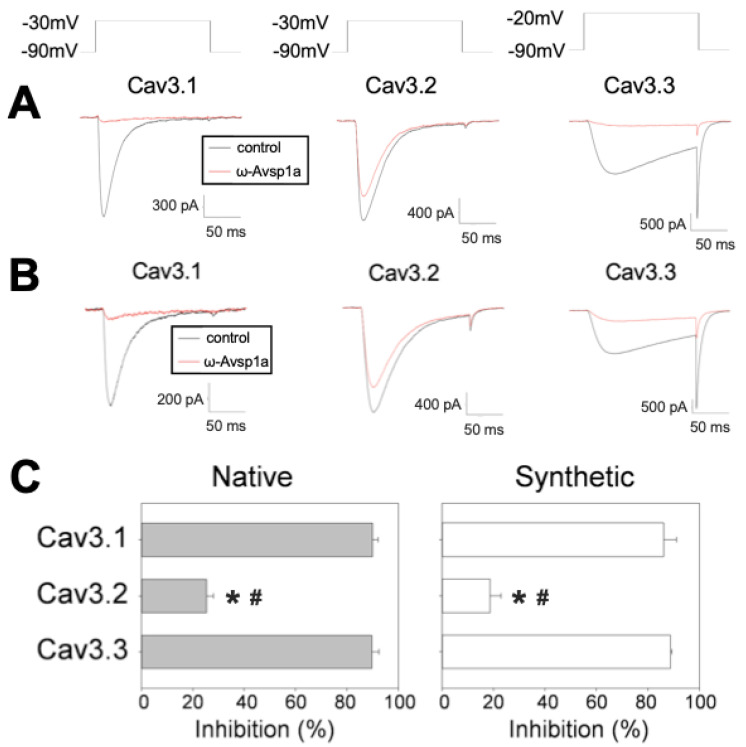
Comparison of the effect of native and synthetic ω-Avsp1a on Ca_V_3.1, Ca_V_3.2, or Ca_V_3.3 expressed in HEK-293 cells. Plasmids of Cav3.1, Cav3.2 or Cav3.3 were separately transfected into HEK-293 cells. (**A**) ω-Avsp1a purified from venom potently inhibited Ca_V_3.1 and Ca_V_3.3 but it was only a weak inhibitor of Ca_V_3.2. The native toxin concentration was 4.1 μM. *n* = 5 for Ca_V_3.1 and Ca_V_3.2 and *n* = 3 for Ca_V_3.3. The voltage-clamp protocols to elicit Cav3.1, Cav3.2 or Cav3.3 are indicated above (**B**) synthetic ω-Avsp1a replicates the inhibitory effects of the native toxin, with potent inhibition of Ca_V_3.1 and Ca_V_3.3 and weak inhibition of Ca_V_3.2. The concentration of synthetic ω-Avsp1a was 10 μM. *n* = 9 for Ca_V_3.1 and Ca_V_3.3, and *n* = 6 for Ca_V_3.2. (**C**) Quantification of the inhibitory effects of native and synthetic ω-Avsp1a on human Ca_V_3 channels. * *p* < 0.001 vs. Cav3.1 and ^#^ *p* < 0.001 vs. Cav3.3 according to one-way ANOVA with Tukey’s post hoc test.

In order to determine the Ca_V_3 selectivity of ω-Avsp1a, we tested native ω-Avsp1a isolated from *A. spec.* venom on Ca_V_3.1, Ca_V_3.2 and Ca_V_3.3 expressed in HEK-293 cells. Native ω-Avsp1a (4.1 μM) inhibited Ca_V_3.1 and Ca_V_3.3 currents by ~90%, but it was much less effective against Ca_V_3.2 (~25% current inhibition) (Figure 4A,C). Similar results were obtained with 10 μM *synthetic* ω-Avsp1a, which inhibited Ca_V_3.1 and Ca_V_3.3 currents by ~90%, but reduced Ca_V_3.2 currents by only ~32% (Figure 4B,C).

### 3.4. Identification of T-Type Calcium Channel α1-Subunit Domains Involved in ω-Avsp1a Activity

**Figure 5:**
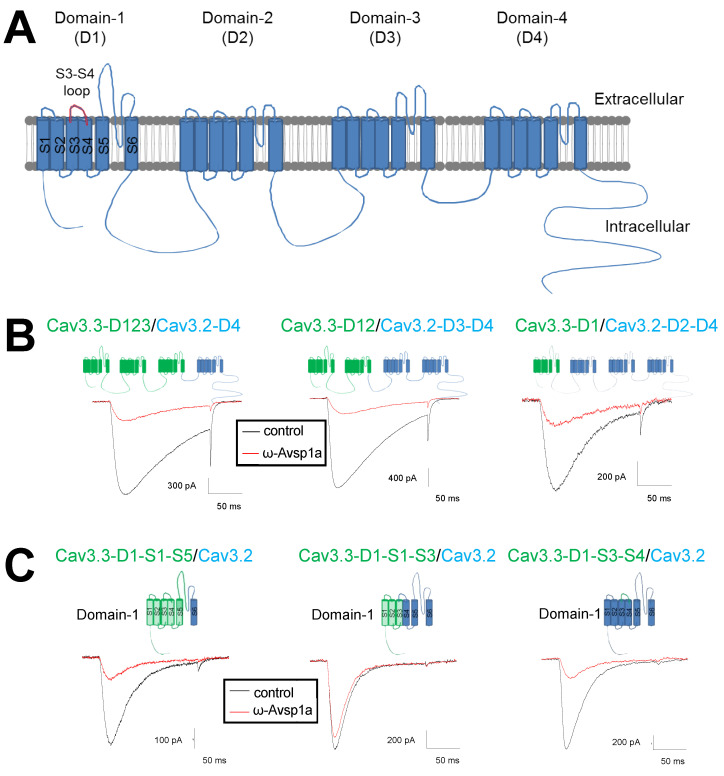
Activity of native ω-Avsp1a on Ca_V_3 chimeras expressed in HEK-293 cells. (**A**) The pore-forming α1-subunit of Ca_V_3 channels is comprised of four homologous domains (D1, D2, D3, D4), each containing six membrane-spanning α-helices (S1–S6) connected by two cytoplasmic and three extracellular loops (S1–S2, S3–S4, S5–S6). The extracellular S3–S4 loop is of particular interest as a well-documented site for binding venom peptides, and it is highlighted in red. (**B**) Identification of domains important for inhibition Ca_V_3 channels by native ω-Avsp1a. The toxin concentration was 4.1 μM. *n* = 3, 3 and 4 for Ca_V_3.3-D1-D3/Ca_V_3.2-D4, Ca_V_3.3-D1-D2/Ca_V_3.2-D3-D4 and Ca_V_3.3-D1/Ca_V_3.2-D2-D4, respectively. (**C**) Identification of domain-1 sub-regions important for inhibition of T-type calcium channel by 4.1 μM native ω-Avsp1a. *n* = 3 for each group.

We assume that the domain structure and amino acid sequence differences between the various Ca_V_3 subtypes underlie their differential sensitivity to ω-Avsp1a. The pore-forming α1-subunit of all Ca_V_ channels, including Ca_V_3.1, Ca_V_3.2 and Ca_V_3.3, is comprised of four homologous domains (D1–D4), with each domain containing six transmembrane segments denoted S1–S6 (Figure 5A). In order to identify the amino acid(s) critical for channel inhibition caused by ω-Avsp1a, we generated chimeric channels from the less inhibited Ca_V_3.2 and more affected Ca_V_3.3. Chimeric channels were constructed by linking the D1, D2, and D3 domains of Ca_V_3.3 with D4 of Ca_V_3.2 (Ca_V_3.3-D1-D3/Ca_V_3.2-D4), D1 and D2 of Ca_V_3.3 with D3 and D4 of Ca_V_3.2 (Ca_V_3.3-D1-D2/Ca_V_3.2-D3-D4) and D1 of Ca_V_3.3 with D2, D3, and D4 of Ca_V_3.2 (Ca_V_3.3-D1/Ca_V_3.2-D2-D4) (Figure 5B). We found that the replacement of domains D1–D3 of Ca_V_3.2 with the homologous domains of Ca_V_3.3 increased the sensitivity of Ca_V_3.2 to ω-Avsp1a to that of Ca_V_3.3 (Figure 5B). We further reduced the number of replaced domains to two (Ca_V_3.3-D1-D2/Ca_V_3.2-D3-D4) or one (Ca_V_3.3-D1/Ca_V_3.2-D2-D4) and found that replacing just D1 of Ca_V_3.2 with the equivalent domain from Ca_V_3.3 is sufficient to enhance the inhibitory effect of ω-Avsp1a on Ca_V_3.2 (Figure 5B). We conclude that domain D1 of Ca_V_3.3 is critical for the inhibitory effect of ω-Avsp1a.

### 3.5. Identification of ω-Avsp1a Binding Site on T-Type Calcium Channels

To narrow down the region within the D1 domain of Ca_V_3.3 required for ω-Avsp1a inhibition, we generated additional chimeric channels. Each of the four homologous domains of Ca_V_ channels contains six transmembrane helices (S1–S6) connected by two cytoplasmic and three extracellular loops (S1–S2, S3–S4 and S5–S6) as shown in Figure 5A. Replacement of D1-S1-S5 of Ca_V_3.2 with the homologous S1–S5 helices of Ca_V_3.3 D1 increased the inhibition induced by ω-Avsp1a from 25% to 71% (see Ca_V_3.3-D1-S1-S5/Ca_V_3.2 in Figure 5C). However, replacement of D1 S1–S3 of Ca_V_3.2 with the equivalent region from Ca_V_3.3 only increased the level of inhibition to 38% (see Ca_V_3.3-D1-S1-S3/Ca_V_3.2 of Figure 5C). These data suggest that the S3–S6 region of Ca_V_3.3 is important for inhibition by ω-Avsp1a. Interestingly, it was previously shown that the D1 S3–S4 extracellular loop of Ca_V_3.2 is important for sensing nickel and reducing agents [28,29]. We therefore replaced the D1 S3–S4 loop of Ca_V_3.2 with the homologous extracellular loop from Ca_V_3.3 and found that it is sufficient to make Ca_V_3.2 as sensitive to ω-Avsp1a as Ca_V_3.3 (see Ca_V_3.3-D1-S3-S4/Ca_V_3.2 in Figure 5C). These results suggest that amino acid residues in the D1 S3–S4 loop of Ca_V_3.3 are critical for the inhibitory effect of ω-Avsp1a.

**Figure 6:**
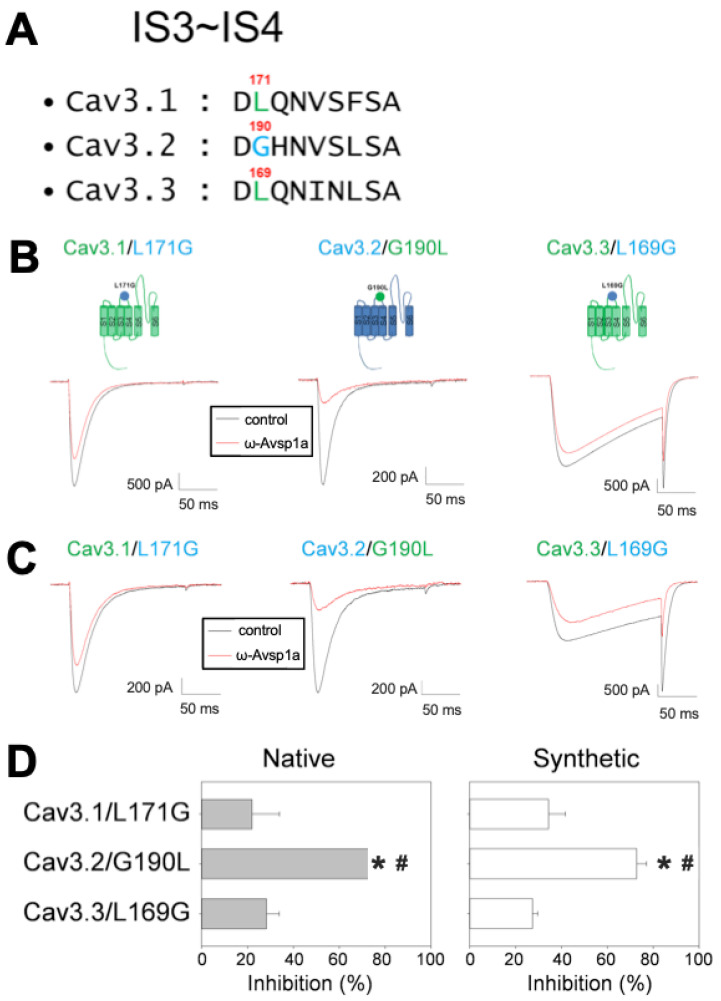
Activity of native and synthetic ω-Avsp1a on Ca_V_3 channel chimeras expressed in HEK-293 cells. (**A**) Alignment of the sequences of the D1 S3-S4 extracellular loops of Ca_V_3.1, Ca_V_3.2 and Ca_V_3.3. The G190 of Ca_V_3.2 is an equivalent position to L171 in Ca_V_3.1and L169 in Ca_V_3.3. (**B**) Identification of residues involved in the inhibitory effect of native ω-Avsp1a. The native toxin concentration was 4.1 μM. *n* = 5 for Ca_V_3.1/L171G and *n* = 4 for Ca_V_3.2/G190L and Ca_V_3.3/L169G. (**C**) Mutations of key residues in Ca_V_3.1, Ca_V_3.2 and Ca_V_3.3 channels reversed the inhibitory effect of synthetic ω-Avsp1a. The concentration of synthetic ω-Avsp1a was 10 μM. *n* = 6, 9 and 4 for Ca_V_3.1/L171G, Ca_V_3.2/G190L and Ca_V_3.3/L169G, respectively. (**D**) Quantification of the inhibitory effect of native and synthetic ω-Avsp1a on the mutant channels. * *p* < 0.001 vs. Cav3.1 and ^#^ *p* < 0.001 vs. Cav3.3 according to one-way ANOVA with Tukey’s post hoc test.

Next, we tried to pinpoint residues within the D1 S3–S4 loop involved in interaction with ω-Avsp1a. Alignment of the D1 S3–S4 loops of Ca_V_3.1–3.3 revealed a high level of similarity, but notably a hydrophobic branched-chain leucine residue at position 169 in Ca_V_3.3 and position 171 in Ca_V_.3.1 is replaced by a small glycine residue at the equivalent position in Ca_V_3.2 (Figure 6A). To examine the role of this leucine residue in the interaction with ω-Avsp1a, we mutated G190 in Ca_V_3.2 to leucine and found that this single mutation markedly increased the toxin sensitivity of Ca_V_3.2 (Figure 6B,D). In contrast, when L171 in Ca_V_3.1 or L169 in Ca_V_3.3 was replaced by glycine, ω-Avsp1a failed to effectively inhibit the mutant channels (Figure 6B,D). Using synthetic rather than native ω-Avsp1a recapitulated these effects (Figure 6C,D). Conversely, the neighbouring glutamine in position 172 does not seem to be involved in toxin binding (Supplementary Figure S2). We therefore conclude that ω-Avsp1a differentially inhibits Ca_V_3 isoforms by making a crucial interaction with a leucine residue in the extracellular D1 S3–S4 loop. Moreover, since ω-Avsp1a modulates the activity of Ca_V_3.1/Ca_V_3.3 by binding to the voltage sensor domain, it is clearly an allosteric modulator of channel activity rather than a pore blocker.

### 3.6. Quantification of the Potency of Synthetic ω-Avsp1a against Ca_V_3.1 and Ca_V_3.3

**Figure 7:**
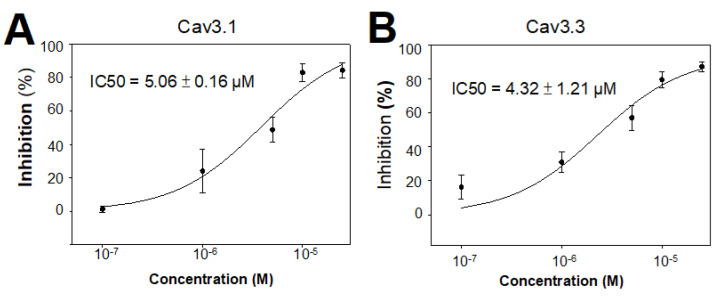
Concentration-response relationship for inhibition of Ca_V_3.1 and Ca_V_3.3 by ω-Avsp1a. Currents mediated by (**A**) Ca_V_3.1 and (**B**) Ca_V_3.3 expressed in HEK-293 cells were recorded before and after treatment with the indicated concentration of synthetic ω-Avsp1a. The IC_50_ values were estimated by fitting a Hill Equation to the data.

We examined the inhibitory potency of a range of ω-Avsp1a concentrations (10, 5, 1, and 0.1 μM) on Ca_V_3.1 (Figure 7A) and Ca_V_3.3 (Figure 7B) in order to quantify the toxin’s potency. Here, only the synthetic ω-Avsp1a was used because of the difficulty of obtaining native toxin from the spider. The calculated IC_50_ values for Ca_V_3.1 and Ca_V_3.3 were 5.06 ± 0.16 and 4.32 ± 1.21 μM, respectively. Because 10 μM ω-Avsp1a inhibited Ca_V_3.2 by only 19%, we increased the concentration to 25 μM, but still only observed 31% inhibition. We were unable to further increase the working concentration of ω-Avsp1a because of limited solubility. Thus, the IC_50_ for ω-Avsp1a inhibition Ca_V_3.2 remains undetermined, but it is >25 μM.

### 3.7. The 3D Structure of ω-Avsp1a

**Figure 8:**
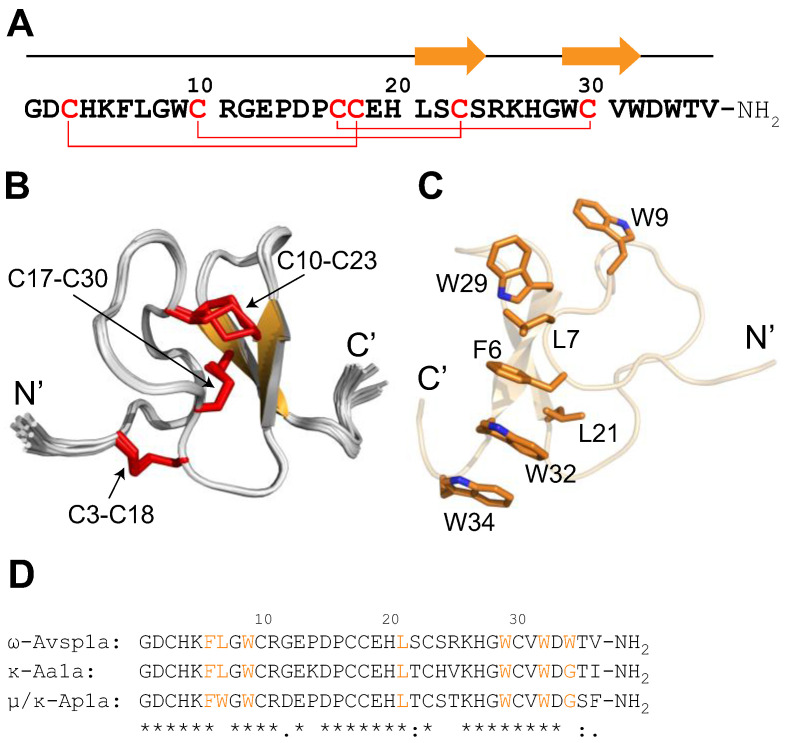
Structure of ω-Avsp1a. (**A**) Primary and secondary structure of ω-Avsp1a. Disulfide bonds are indicated by red lines and the secondary structure of the peptide is shown above the sequence, with orange arrows representing β-strands. (**B**) Ensemble of 20 ω-Avsp1a structures determined using solution-state NMR spectroscopy. β-strands are orange and disulfide bonds are indicated by red tubes. The N- and C-termini and disulfide bonds are labelled. (**C**) Structure of ω-Avsp1a highlighting the ladder of hydrophobic residues. The sidechains of the seven residues that contribute to this hydrophobic ladder (F6, L7, W9, L21, W29, W32 and W34) are shown as orange tubes. (**D**) Sequence alignment of the three C-terminally amidated *Avicularia* toxins ω-Avsp1a, κ-Aa1a and μ/κ-Ap1a made using CLUSTAL Omega 1.2.4 (www.ebi.ac.uk/Tools/msa/clustalo/; accessed on 1 May 2022). The seven hydrophobic residues specified in (**C**) are highlighted in orange in (**D**). Amino acid sequence conservation is indicated by (*) = fully conserved residues, (:) = conservation of residues with strongly similar properties, (.) = conservation of residues with weakly similar properties.

The solution structure of ω-Avsp1a was determined using NMR spectroscopy. We obtained chemical shift assignments for 99.5% of the proton resonances, and the structure was calculated based on 565 interproton-distance restraints, 52 dihedral-angle restraints and 9 disulfide-bond restraints. The final ensemble of 20 structures is highly precise, with a root-mean-square deviation of 0.16 ± 0.06 Å over the backbone atoms of residues 3–34, which excludes the flexible N- and C-terminal regions. The peptide conforms to the inhibitor cystine knot (ICK) motif which is commonly found in spider-venom peptides [30,31]. This motif is recognisable by three disulfide bonds with C1–C4, C2–C5, C3–C6 connectivity [32] that form a “pseudo-knot” architecture in which two of the disulfide bonds and the intervening sections of the peptide backbone form a ring that is pierced by the third disulfide bond (Figure 8). In addition to the core cystine knot, the ICK motif is characterised by an antiparallel β sheet which in ω-Avsp1a is formed by residues L21–S24 (β1) and W29–W32 (β2). Interestingly, there is a highly ordered network of hydrophobic residues adjacent to β2 which includes all of the aromatic (F6, W9, W29, W32, W34) and leucine (L7, L21) residues in the peptide. These hydrophobic residues stack like the rungs of a ladder on one face of the peptide, whereas the charged residues, in contrast, are more evenly distributed over the surface of the peptide.

## 4. Discussion

ω-Avsp1a, the dominant component of *Avicularia spec.* (“Amazonas Purple”, Peru) venom, was initially isolated from the venom based on its inhibition of Ca_V_3. Edman sequencing revealed only the N-terminal 30 residues of the peptide, while the remaining six C-terminal residues were determined via MALDI-TOF MS following digestion of the peptide with CPY. Together, the Edman sequencing and MS data suggested the following amino acid sequence for ω-Avsp1a: GDCHKFLGWCRGEPDPCCEHLSCSRKHGWCVWDWTV-NH_2_. Given that (i) synthetic ω-Avsp1a corresponding to this sequence co-elutes with native ω-Avsp1a on RP-HPLC; (ii) the molecular mass predicted from this sequence matches that of native ω-Avsp1a; and (iii) the activity of synthetic ω-Avsp1a on the various Ca_V_3 subtypes recapitulates that of the native peptide, we are confident that the sequence we determined for ω-Avsp1a is correct.

NMR analysis revealed that the structure of ω-Avsp1a conforms to the ICK motif, which is commonly found in spider-venom peptides. The ICK architecture endows peptides with resistance to proteases, harsh chemicals, and high temperatures [33], which generally provides them with high levels of stability in human serum [34,35,36]. This property makes this class of peptide an attractive source of peptide-drug leads. The structure of ω-Avsp1a revealed a narrow hydrophobic strip composed of a stack of aromatic and leucine residues. Such hydrophobic patches are commonly found in spider toxins that modulate ion channel activities via interaction with one or more of the voltage-sensing domains of the channel. These hydrophobic patches have been proposed to facilitate interaction of the toxin with the plasma membrane so that it can diffuse laterally within the lipid bilayer to interact with the membrane-embedded voltage-sensor domain [37,38].

Interestingly, a similar distribution of hydrophobic residues (with only W34 missing; see Figure 8D) is present in two other *Avicularia* toxins (κ-Aa1a and μ/κ-Ap1a) that inhibit the human ether-à go-go voltage-gated potassium channels hEAG1 (K_V_10.1). ω-Avsp1a is 83.3% identical to κ-Aa1a and 80.6% identical to μ/κ-Ap1a [39]. Thus, it would be interesting in future studies to determine whether ω-Avsp1a targets Kv10.1 and whether κ-Aa1a and μ/κ-Ap1a are active on Ca_V_3 channels.

It is well documented that the three Ca_V_3 subtypes participate differentially in a variety of physiological and pathological conditions. Selective pharmacological inhibition is therefore important for dissecting the pathophysiological functions of Ca_V_3 channel subtypes and for the development of therapeutically useful subtype-selective inhibitors. Ca_V_3 inhibitors such as ethosuximide and zonisamide are used clinically as anti-epileptic drugs, but they are not subtype-specific and not effective in all patients [40]. Preclinical data suggest that Ca_V_3.2 might be a viable analgesic target, but none of the Ca_V_3 blockers that have advanced to human clinical trials have been successful [41]. Thus, there is urgent need to identify new subtype-selective Ca_V_3 inhibitors. In this study, we showed that ω-Avsp1a has 10-fold or higher selectivity for Ca_V_3.1/Ca_V_3.3 over Ca_V_3.2, suggesting that it might be a useful pharmacological tool for dissecting out the role of Ca_V_3 channels in various human pathophysiologies and for developing selective inhibitors of Ca_V_3.1/Ca_V_3.3.

Our chimeric channel experiments clearly demonstrate that ω-Avsp1a interacts with the extracellular S3–S4 loop of Ca_V_3 and that Leu171 in this loop is essential for its inhibitory activity. Replacement of this Leu residue by Gly, the amino acid in the homologous position of Ca_V_3.2, massively diminished the inhibitory activity of the peptide. The structure of ω-Avsp1a determined using NMR spectroscopy revealed a narrow hydrophobic strip, which is likely to be the region of the toxin that mediates its interaction with Leu171 in Ca_V_3.1 and Leu169 in Ca_V_3.3. In the Cryo-EM structure of Ca_V_3.1 [42], an α-helix formed by the sequence around Leu171 of Ca_V_3.1 is located in the most extracellular portion of the channel, providing a possible site for interaction with ω-Avsp1a. Our data suggest that targeting the D1 S3–S4 loop of Ca_V_3 channels might be a good strategy for developing subtype-selective inhibitors of these channels.

## 5. Conclusions

A bioassay-guided fractionation strategy was used to isolate peptide ω-Avsp1a from venom of the Peruvian tarantula *Avicularia spec.* (“Amazonas Purple”). ω-Avsp1a was found to be a selective inhibitor of Ca_V_3.1 and Ca_V_3.3, with at least five-fold weaker activity on Ca_V_3.2. By engineering chimeric Ca_V_ channel constructs, we narrowed the ω-Avsp1a binding site on Ca_V_3.3 to the D1 S3–S4 extracellular loop and showed that Leu171 in this loop is crucial for the toxin–channel interaction. Despite the low potency of ω-Avsp1a, its selectivity for Ca_V_3.1/Ca_V_3.3 over Ca_V_3.2 makes it a useful pharmacological tool for dissecting out the role of these different Ca_V_3 subtypes in disorders such as epilepsy or pain.

## Figures and Tables

**Table 1:** Structural statistics for the NMR ensemble of ω-Avsp1a ^a^.

PDB ID	7LVN
Experimental restraints	
Inter-proton distance restraints	
Total	565
Intra-residue (*i = j*)	137
Sequential (|*i − j|* = 1)	178
Medium range (1 < |*i − j|* < 5)	88
Long range (|*i − j|* ≥ 5)	162
Disulfide bond restraints	9
Dihedral-angle restraints	52
φ dihedral angle restraints	23
ψ dihedral angle restraints	21
χ^1^ angle restraints	8
Total number of restraints per residue	17.4
Violations of experimental restraints	0
RMSD from mean coordinate structure (Å) ^b^	
All backbone atoms	0.43 ± 0.10
All heavy atoms	0.74 ± 0.12
Backbone atoms (Residue 3–34)	0.16 ± 0.06
Heavy atoms (Residue 3–34)	0.63 ± 0.11
Stereochemical quality ^c^	
Ramachandran plot statistics	
Residues in most favoured Ramachandran region (%)	76.0 ± 2.6
Disallowed regions [%]	2.4 ± 1.2
Unfavourable sidechain rotamers [%]	7.8 ± 1.9
Clashscore, all atoms ^d^	0.0 ± 0.0
Overall MolProbity score	1.95 ± 0.09

^a^ All statistics are given as mean ± S.D; ^b^ Mean RMSD calculated over the entire ensemble of 20 structures; ^c^ Sterochemical quality according to MolProbity (http://helix.research.duhs.duke.edu, accessed on 9 February 2022); ^d^ Clashscore is defined as the number of steric overlaps > 0.4 Å per 1000 atoms.

## Data Availability

Not applicable.

## Supplementary Materials

The following supporting information can be downloaded at: https://www.mdpi.com/article/10.3390/biomedicines10051066/s1. Table S1: Crude arachnid venoms screened by patch-clamp electrophysiology for inhibitory activity (indicated in percent inhibition) against three Ca_V_3 subtypes. n.d. = not determined. The venom of the Avicularia spec. that was used for the isolation of ω-Avsp1a is highlighted in red. Figure S1: Screening for Ca_V_3 channel subtype specific inhibitors from arachnid venoms. Figure S2: Q172 of Ca_V_3.1 is not important for inhibition function of ω-Avsp1a. Ca_V_3.1/Q172 was mutated to histidine as the homologous residue in Ca_V_3.2. There is no statistically significant difference between Ca_V_3.1 and Ca_V_3.1/Q172H mutant (Ca_V_3.1 vs Ca_V_3.1/Q172H, P = 0.70 according to two-tailed T-test). n = 5, 3, 5, 3, for Ca_V_3.1, Ca_V_3.1/Q172H, Ca_V_3.2, Ca_V_3.2/H191Q, respectively.

## References

[1] Missiaen L., Callewaert G., Parys J.B., Wuytack F., Raeymaekers L., Droogmans G., Nilius B., Eggermont J., De Smedt H. (2000). Intracellular calcium: Physiology and physiopathology. Verh.-K. Acad. Geneeskd. Belg..

[2] Missiaen L., Robberecht W., van den Bosch L., Callewaert G., Parys J.B., Wuytack F., Raeymaekers L., Nilius B., Eggermont J., De Smedt H. (2000). Abnormal intracellular Ca^2+^homeostasis and disease. Cell Calcium.

[3] Perez-Reyes E. (2003). Molecular physiology of low-voltage-activated T-type calcium channels. Physiol. Rev..

[4] Kim D., Song I., Keum S., Lee T., Jeong M.J., Kim S.S., McEnery M.W., Shin H.S. (2001). Lack of the burst firing of thalamocortical relay neurons and resistance to absence seizures in mice lacking α(1G) T-type Ca^2+^ channels. Neuron.

[5] Chen C.C., Lamping K.G., Nuno D.W., Barresi R., Prouty S.J., Lavoie J.L., Cribbs L.L., England S.K., Sigmund C.D., Weiss R.M. (2003). Abnormal coronary function in mice deficient in α1H T-type Ca^2+^ channels. Science.

[6] Tzeng B.H., Chen Y.H., Huang C.H., Lin S.S., Lee K.R., Chen C.C. (2012). The Ca_V_3.1 T-type calcium channel is required for neointimal formation in response to vascular injury in mice. Cardiovasc. Res..

[7] Mangoni M.E., Traboulsie A., Leoni A.L., Couette B., Marger L., Le Quang K., Kupfer E., Cohen-Solal A., Vilar J., Shin H.S. (2006). Bradycardia and slowing of the atrioventricular conduction in mice lacking Ca_V_3.1/α1G T-type calcium channels. Circ. Res..

[8] Anderson M.P., Mochizuki T., Xie J., Fischler W., Manger J.P., Talley E.M., Scammell T.E., Tonegawa S. (2005). Thalamic Ca_V_3.1 T-type Ca^2+^ channel plays a crucial role in stabilizing sleep. Proc. Natl. Acad. Sci. USA.

[9] Lee J., Kim D., Shin H.S. (2004). Lack of delta waves and sleep disturbances during non-rapid eye movement sleep in mice lacking α1G-subunit of T-type calcium channels. Proc. Natl. Acad. Sci. USA.

[10] Chiang C.S., Huang C.H., Chieng H., Chang Y.T., Chang D., Chen J.J., Chen Y.C., Chen Y.H., Shin H.S., Campbell K.P. (2009). The Ca_V_3.2 T-type Ca^2+^ channel is required for pressure overload-induced cardiac hypertrophy in mice. Circ. Res..

[11] Bourinet E., Alloui A., Monteil A., Barrere C., Couette B., Poirot O., Pages A., McRory J., Snutch T.P., Eschalier A. (2005). Silencing of the Ca_V_3.2 T-type calcium channel gene in sensory neurons demonstrates its major role in nociception. EMBO J..

[12] Garcia-Caballero A., Gadotti V.M., Stemkowski P., Weiss N., Souza I.A., Hodgkinson V., Bladen C., Chen L., Hamid J., Pizzoccaro A. (2014). The deubiquitinating enzyme USP5 modulates neuropathic and inflammatory pain by enhancing Ca_V_3.2 channel activity. Neuron.

[13] Chen W.K., Liu I.Y., Chang Y.T., Chen Y.C., Chen C.C., Yen C.T., Shin H.S., Chen C.C. (2010). Ca_V_3.2 T-type Ca^2+^ channel-dependent activation of ERK in paraventricular thalamus modulates acid-induced chronic muscle pain. J. Neurosci..

[14] Hansen P.B. (2015). Functional importance of T-type voltage-gated calcium channels in the cardiovascular and renal system: News from the world of knockout mice. Am. J. Physiol. Regul. Integr. Comp. Physiol..

[15] Nakayama H., Bodi I., Correll R.N., Chen X.W., Lorenz J., Houser S.R., Robbins J., Schwartz A., Molkentin J.D. (2009). α1G-dependent T-type Ca^2+^ current antagonizes cardiac hypertrophy through a NOS3-dependent mechanism in mice. J. Clin. Investig..

[16] Bourinet E., Zamponi G.W. (2017). Block of voltage-gated calcium channels by peptide toxins. Neuropharmacology.

[17] Norton R.S., McDonough S.I. (2008). Peptides targeting voltage-gated calcium channels. Curr. Pharm. Des..

[18] Cardoso F.C. (2020). Multi-targeting sodium and calcium channels using venom peptides for the treatment of complex ion channels-related diseases. Biochem. Pharmacol..

[19] King G.F. (2007). Modulation of insect Ca_V_ channels by peptidic spider toxins. Toxicon.

[20] Pringos E., Vignes M., Martinez J., Rolland V. (2011). Peptide neurotoxins that affect voltage-gated calcium channels: A close-up on ω-agatoxins. Toxins.

[21] Herzig V., Hodgson W.C. (2009). Intersexual variations in the pharmacological properties of *Coremiocnemis tropix* (Araneae, Theraphosidae) spider venom. Toxicon.

[22] Osteen J.D., Herzig V., Gilchrist J., Emrick J.J., Zhang C., Wang X., Castro J., Garcia-Caraballo S., Grundy L., Rychkov G.Y. (2016). Selective spider toxins reveal a role for the Na_V_1.1 channel in mechanical pain. Nature.

[23] Vranken W.F., Boucher W., Stevens T.J., Fogh R.H., Pajon A., Llinas M., Ulrich E.L., Markley J.L., Ionides J., Laue E.D. (2005). The CCPN data model for NMR spectroscopy: Development of a software pipeline. Proteins.

[24] Güntert P., Buchner L. (2015). Combined automated NOE assignment and structure calculation with CYANA. J. Biomol. NMR.

[25] Shen Y., Bax A. (2013). Protein backbone and sidechain torsion angles predicted from NMR chemical shifts using artificial neural networks. J. Biomol. NMR.

[26] Williams C.J., Headd J.J., Moriarty N.W., Prisant M.G., Videau L.L., Deis L.N., Verma V., Keedy D.A., Hintze B.J., Chen V.B. (2018). MolProbity: More and better reference data for improved all-atom structure validation. Protein Sci..

[27] King G.F., Gentz M.C., Escoubas P., Nicholson G.M. (2008). A rational nomenclature for naming peptide toxins from spiders and other venomous animals. Toxicon.

[28] Kang H.W., Park J.Y., Jeong S.W., Kim J.A., Moon H.J., Perez-Reyes E., Lee J.H. (2006). A molecular determinant of nickel inhibition in Ca_V_3.2 T-type calcium channels. J. Biol. Chem..

[29] Nelson M.T., Woo J., Kang H.W., Vitko I., Barrett P.Q., Perez-Reyes E., Lee J.H., Shin H.S., Todorovic S.M. (2007). Reducing agents sensitize C-type nociceptors by relieving high-affinity zinc inhibition of T-type calcium channels. J. Neurosci..

[30] King G.F., Hardy M.C. (2013). Spider-venom peptides: Structure, pharmacology, and potential for control of insect pests. Annu. Rev. Entomol..

[31] Pineda S.S., Chin Y.K., Undheim E.A.B., Senff S., Mobli M., Dauly C., Escoubas P., Nicholson G.M., Kaas Q., Guo S. (2020). Structural venomics reveals evolution of a complex venom by duplication and diversification of an ancient peptide-encoding gene. Proc. Natl. Acad. Sci. USA.

[32] Norton R.S., Pallaghy P.K. (1998). The cystine knot structure of ion channel toxins and related polypeptides. Toxicon.

[33] Herzig V., King G.F. (2015). The cystine knot is responsible for the exceptional stability of the insecticidal spider toxin ω-Hexatoxin-Hv1a. Toxins.

[34] Chow C.Y., Chin Y.K.Y., Ma L., Undheim E.A.B., Herzig V., King G.F. (2020). A selective Na_V_1.1 activator with potential for treatment of Dravet syndrome epilepsy. Biochem. Pharmacol..

[35] Ma L., Chin Y.K.Y., Dekan Z., Herzig V., Chow C.Y., Heighway J., Lam S.W., Guillemin G.J., Alewood P.F., King G.F. (2018). Novel venom-derived inhibitors of the human EAG channel, a putative antiepileptic drug target. Biochem. Pharmacol..

[36] Redd M.A., Scheuer S.E., Saez N.J., Yoshikawa Y., Chiu H.S., Gao L., Hicks M., Villanueva J.E., Joshi Y., Chow C.Y. (2021). Therapeutic inhibition of acid-sensing ion channel 1a recovers heart function after ischemia-reperfusion injury. Circulation.

[37] Deplazes E., Henriques S.T., Smith J.J., King G.F., Craik D.J., Mark A.E., Schroeder C.I. (2016). Membrane-binding properties of gating modifier and pore-blocking toxins: Membrane interaction is not a prerequisite for modification of channel gating. Biochim. Biophys. Acta.

[38] Henriques S.T., Deplazes E., Lawrence N., Cheneval O., Chaousis S., Inserra M., Thongyoo P., King G.F., Mark A.E., Vetter I. (2016). Interaction of tarantula venom peptide ProTx-II with lipid membranes Is a prerequisite for its inhibition of human voltage-gated sodium channel Na_V_1.7. J. Biol. Chem..

[39] Huang X., Miller W. (1991). A time-efficient, linear-space local similarity algorithm. Adv. Appl. Math..

[40] Powell K.L., Cain S.M., Snutch T.P., O’Brien T.J. (2014). Low threshold T-type calcium channels as targets for novel epilepsy treatments. Br. J. Clin. Pharmacol..

[41] Snutch T.P., Zamponi G.W. (2018). Recent advances in the development of T-type calcium channel blockers for pain intervention. Br. J. Pharmacol..

[42] Zhao Y., Huang G., Wu Q., Wu K., Li R., Lei J., Pan X., Yan N. (2019). Cryo-EM structures of apo and antagonist-bound human Cav3.1. Nature.

