# Supplementary material for: The Tarantula Toxin ω-Avsp1a Specifically Inhibits Human CaV3.1 and CaV3.3 via the Extracellular S3-S4 Loop of the Domain 1 Voltage-Sensor
Source: Biomedicines. 2022 May 4;10(5):1066. doi: 10.3390/biomedicines10051066 (PMC9138389; doi:10.3390/biomedicines10051066)
Supplement: Supplementary file 1 [file biomedicines-10-01066-s001.zip › biomedicines-1679530-supplementary.pdf]

Supplementary

The tarantula toxin ω-Avsp1a specifically inhibits human CaV3.1 and CaV3.3 via the extracellular S3-S4 loop of the domain 1 voltage-sensor

Volker Herzig[1,2,3, §,*], Yong-Cyuan Chen[4, §], Yanni K.-Y. Chin[1,5], Zoltan Dekan[1], Yu-Wang Chang[4], Hui-Ming Yu[6], Paul F. Alewood[1], Chien-Chang Chen[4*], and Glenn F. King[1,7,*]

[1] Institute for Molecular Bioscience, The University of Queensland, Brisbane QLD 4072, Australia
[2] Centre for Bioinnovation; University of the Sunshine Coast, Sippy Downs, QLD 4556, Australia
[3] School of Science, Technology and Engineering, University of the Sunshine Coast, Sippy Downs, QLD 4556, Australia
[4] Institute of Biomedical Sciences, Academia Sinica, Taipei 11529, Taiwan
[5] Centre for Advanced Imaging, The University of Queensland, Brisbane QLD 4072, Australia
[6] Genomics Research Center, Academia Sinica, Taipei 11529, Taiwan
[7] Australian Research Council Centre of Excellence for Innovations in Peptide and Protein Science, The University of Queensland, St Lucia, QLD 4072, Australia
§ Equal contribution
* Correspondence: A/Prof. Volker Herzig, vherzig@usc.edu.au; Tel.: +61-7-5456-5382; Prof. Chien-Chang Chen, ccchen@ibms.sinica.edu.tw; Tel.: 886-2-2652-3522; Prof. Glenn F. King, glenn.king@imb.uq.edu.au; Tel.: +61-7-3346-2025

Venom Screen

In order to identify Cav3 subtype-specific inhibitors, human Cav3.1, Cav3.2 or Cav3.3 were separately transfected into HEK-293 cells. Each crude venom was applied to HEK-293 cells expressed solely Cav3.1, Cav3.2 or Cav3.3. The Cav3 subtypes inhibition effects of crude venoms were then determined by voltage clamp electrophysiology (see sections 2.7 and 2.8 of the main manuscript for details). Overall, 26 spider and one scorpion venoms were screened for activity against all three Ca_V3 subtypes using 400 ng of venom per cell. The results of the venom screening are detailed in the Supplementary Table 1.

Venom from A. spec.("purple") was identified as showing Cav3 subtype-specific inhibition and was therefore separated by RP-HPLC into 60 fractions. Every 5 RP-HPLC fractions were combined and the Cav3 inhibition function of each pool was determined by voltage clamp (Supplementary Figure 1). The active pool was determined and then the active RP-HPLC fraction was identified during a subsequent round of screening. Only one active RP-HPLC fraction was identified and then further purified using HILIC-HPLC (Supplementary Figure 1).

Supplementary Table S1: Crude arachnid venoms screened by patch-clamp electrophysiology for inhibitory activity (indicated in percent inhibition) against three Ca_V3 subtypes. n.d. = not determined. The venom of the *Avicularia spec.* that was used for the isolation of ω-Avsp1a is highlighted in red.

**Organism**	**Family**	**Subfamily**	***Genus***	***Species***	**Sex**	**Cav3.1**	**Cav3.2**	**Cav3.3**
scorpion	Buthidae		*Parabuthus*	*villosus*	n.d.	33.1	45.4	30.9
spider	Araneidae		*Nephila*	pilipes	f	8.0	18.3	15.4
spider	Ctenidae		*Ancylometes*	*spec.(Guatemala)*	f	-4.7	8.2	8.9
spider	Ctenidae		*Phoneutria*	*fera*	f	-1.7	37.0	11.6
spider	Gradungulidae		Hickmania	troglodytes	f	7.9	7.2	13.6
spider	Macrothelidae		*Macrothele*	*gigas*	f	2.2	22.6	1.8
spider	Sparassidae		Neosparassus	diana	f	-6.8	11.0	4.7
spider	Theraphosidae	Aviculariinae	*Avicularia*	*spec. ("purple")*	f	79.2	14.0	64.1
spider	Theraphosidae	Eumenophorinae	*Hysterocrates*	*gigas*	f	26.2	44.4	44.7
spider	Theraphosidae	Eumenophorinae	*Monocentropus*	*lambertoni*	f	36.5	52.5	40.1
spider	Theraphosidae	Harpactirinae	*Ceratogyrus*	*darlingi*	f	66.5	31.9	60.7
spider	Theraphosidae	Harpactirinae	*Pterinochilus*	*murinus*	f	56.6	43.2	93.4
spider	Theraphosidae	Ischnocolinae	*Holothele*	*spec. (Colombia)*	f	5.3	45.8	19.5
spider	Theraphosidae	Ornithoctoninae	*Haplopelma*	*hainanum*	f	19.0	1.4	36.2
spider	Theraphosidae	Poecilotheriinae	*Poecilotheria*	*metallica*	f	10.1	10.3	36.1
spider	Theraphosidae	Poecilotheriinae	*Poecilotheria*	*rufilata*	f	25.6	14.2	62.1
spider	Theraphosidae	Psalmopoeinae	*Psalmopoeus*	*pulcher*	f	-1.0	5.6	48.2
spider	Theraphosidae	Selenocosmiinae	*Chilobrachys*	*dyscolus*	f	13.3	6.9	33.8
spider	Theraphosidae	Selenocosmiinae	*Selenocosmia*	*aruana*	f	3.0	5.4	8.5
spider	Theraphosidae	Stromatopelminae	*Stromatopelma*	*calceatum*	f	52.9	9.8	80.2
spider	Theraphosidae	Theraphosinae	*Acanthoscurria*	*musculosa*	f	14.4	40.0	28.4
spider	Theraphosidae	Theraphosinae	*Grammostola*	*rosea*	f	66.5	47.0	45.9
spider	Theraphosidae	Theraphosinae	*Lasiodora*	*klugi*	f	n.d.	38.5	60.7
spider	Theraphosidae	Theraphosinae	*Pamphobeteus*	*antinous*	f	14.6	20.9	46.4
spider	Theraphosidae	Theraphosinae	*Sericopelma*	*spec. (Panama)*	f	17.5	6.6	29.1
spider	Theraphosidae	Theraphosinae	*Theraphosa*	*apophysis*	f	4.7	12.2	42.3
spider	Theraphosidae	Theraphosinae	*Xenesthis*	*spec. (Colombia)*	f	52.0	55.8	70.9

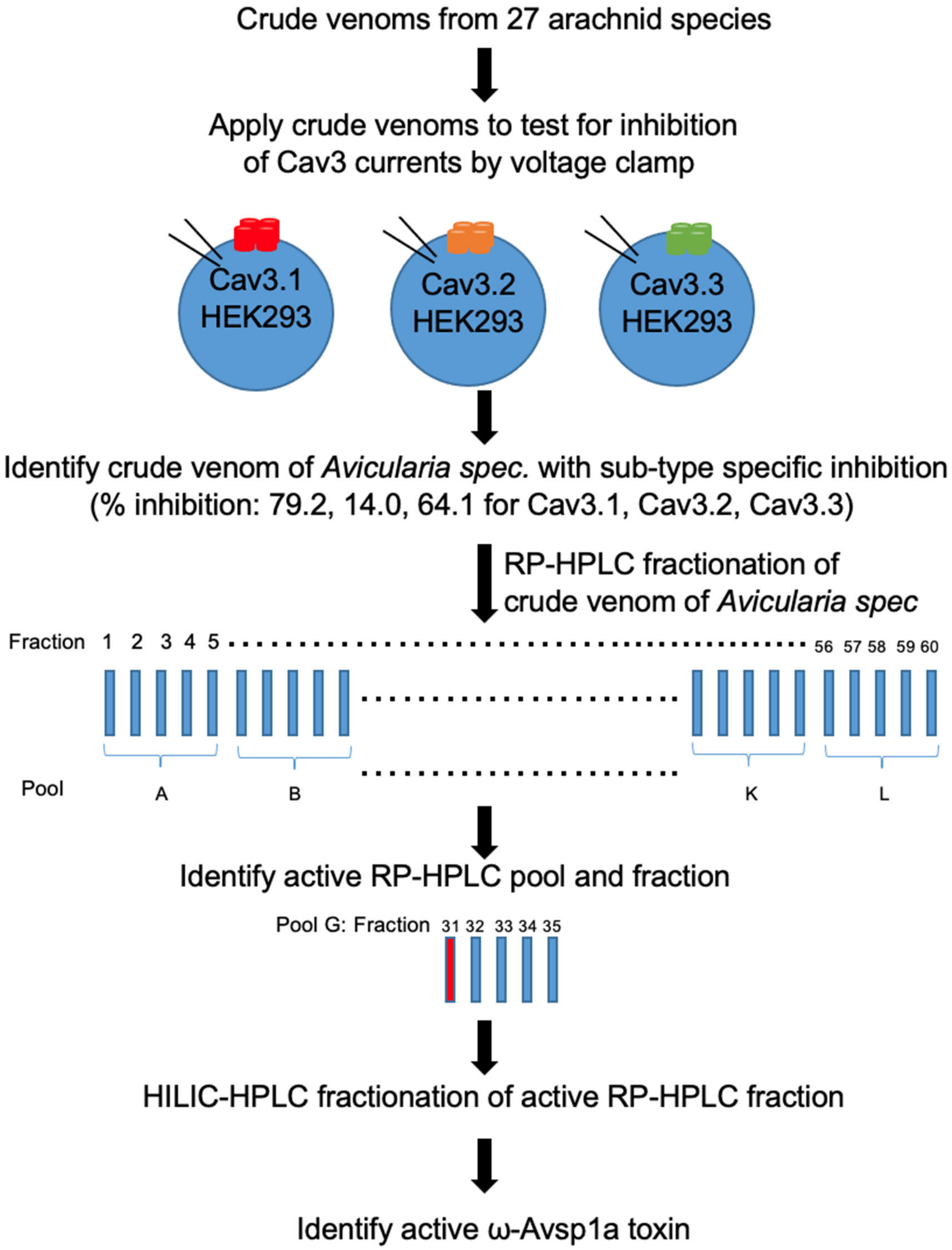

Supplementary Figure S1: Screening for Cav3 channel subtype specific inhibitors from arachnid venoms

Mutation of Cav3.1 residue 172

To determine whether the residue 172, which is different between Cav3.1 (Q), Cav3.2- (H) and Cav3.3 (Q) also plays a role for binding of ω-Avsp1a, we constructed a Cav3.1/Q172H mutant and for the homologous position in Cav3.2 we constructed a Cav3.2/H191Q mutant. Construction of the mutants and electrophysiological assays were performed according to the methods described in sections 2.7-2.9 of the main article). The inhibitory activity of ω-Avsp1a was found to be similar between Cav3.1 and the Cav3.1/Q172H mutant and between Cav3.2 and the Cav3.2/H191Q mutant, respectively (Supplementary Figure 2). In conclusion, residue 172 does not play a role for the interaction with ω-Avsp1a.

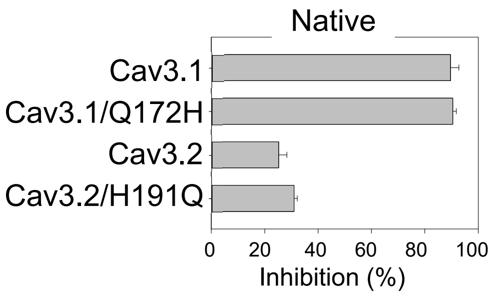

Supplementary Figure S2:

Q172 of Cav3.1 is not important for inhibition function of ω-Avsp1a. Cav3.1/Q172 was mutated to histidine as the homologous residue in Cav3.2. There is no statistically significant difference between Cav3.1 and Cav3.1/Q172H mutant (Cav3.1 vs Cav3.1/Q172H, $P=0.70$ according to two-tailed T-test). $n=5, 3, 5, 3$, for Cav3.1, Cav3.1/Q172H, Cav3.2, Cav3.2/H191Q, respectively.
